# Highly pathogenic avian influenza A(H5N1) in poultry and domestic cats and occupational exposure among veterinary and other first responders, Germany, February 2026

**DOI:** 10.2807/1560-7917.ES.2026.31.17.2600293

**Published:** 2026-04-30

**Authors:** Aparna Dressler, Christiane Wagner-Wiening, Bettina Tegtmeyer, Susanne Haag-Milz, Bettina Demattio, Ralf Dürrwald, Timm Harder, Andreas Salditt, Judith Köster

**Affiliations:** 1 Department of Health Protection, Infection Control and Epidemiology, State Health Office, Ministry of Social Affairs, Health and Integration, Stuttgart, Germany; 2 Heidelberg Institute of Global Health (HIGH), Medical Faculty and University Hospital, Heidelberg University, Heidelberg, Germany; 3 Public Health Office Sigmaringen, Sigmaringen, Germany; 4 Local Veterinary Office Sigmaringen, Sigmaringen, Germany; 5 Robert Koch Institute, Department of infectious diseases, Unit17 Influenza and other respiratory viruses, National Influenza Centre, Berlin, Germany; 6 Institute of Diagnostic Virology, Friedrich-Loeffler Institute, Greifswald-Insel Riems, Germany; 7 Aulendorf State Veterinary Diagnostic Centre, Aulendorf, Germany

**Keywords:** Avian Influenza, HPAI A(H5N1), Zoonotic transmission, Occupational exposure, One Health, Serology, Outbreak, surveillance, Domestic cats, Risk assessment

## Abstract

In February 2026, a highly pathogenic avian influenza (HPAI) A(H5N1) outbreak in a poultry holding in Sigmaringen, Germany, affected poultry and domestic cats, with infection confirmed by RT-qPCR. A One Health investigation identified 17 exposed humans, one of whom developed respiratory symptoms but tested negative for HPAI A(H5N1) (human coronavirus OC43 detected). Serological testing used haemagglutination inhibition assays. This outbreak highlights zoonotic risk, mammalian spillover and the need for coordinated veterinary and public health response and preventive measures.

**Figure:**
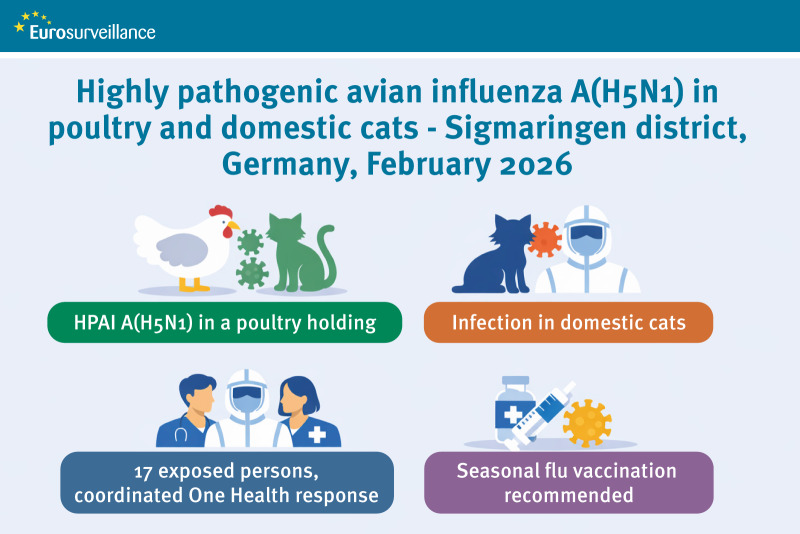


Highly pathogenic avian influenza (HPAI) viruses continue to circulate in Europe, causing outbreaks in poultry and wild birds and occasionally infecting mammals [1-4]. Although human infections remain rare, zoonotic transmission is a recognised occupational risk for persons involved in animal husbandry, outbreak control, and veterinary response activities, and sporadic human infections with HPAI A(H5N1) have been reported globally [5]. An HPAI outbreak in poultry and cats in a small, remote poultry holding in Sigmaringen in February 2026 triggered a One Health investigation with 17 exposed persons. 

Here we describe the outbreak, assess potential zoonotic transmission and evaluate the public health response within a One Health framework.

## Outbreak detection and initial investigation

On 19 February 2026, the local public health authority in Sigmaringen, Baden-Wuerttemberg, Germany, was notified of a suspected avian influenza outbreak in a small poultry holding following veterinary inspections triggered by animal welfare concerns. The holding, which had no biosecurity measures, comprised ca 21 chickens and nine free-roaming cats and was located in a remote rural area. The birds were in a poultry house but had access to the outside and contact with wild birds. 

Between 16 and 18 February, veterinary inspectors found four dead chickens and one dead cat on the premises. A further cat showing severe neurological symptoms was euthanised (Tables 1 and 2). Laboratory testing using real-time quantitative PCR (RT-qPCR) confirmed HPAI A(H5N1) infection in all six animals, poultry and cats. All remaining poultry (n = 17) were culled as part of control measures. Subsequently, an additional symptomatic cat tested PCR-positive and was euthanised. The PCR-positive symptomatic cats presented with diverse clinical manifestations, including neurological signs, respiratory symptoms and general sickness.

**Table 1:** Timeline of highly pathogenic avian influenza A(H5N1) outbreak investigation and monitoring of exposed persons, Sigmaringen, Baden-Wuerttemberg, Germany, February–March 2026

Date	Timeline of events	Contact person group
16 Feb	First on-site inspection by veterinary authority due to animal welfare concerns. One sick cat found and euthanised. Four dead chickens identified on the premises.	V, H, T
17 Feb	Dead cat found on premises	V, P, H
18 Feb	Four dead chickens suspected HPAI	V, H
19 Feb	Public health authority notified. Three cats transferred to animal shelter. Remaining poultry (n = 17) culled. None of the contact persons had any PPE until 18 February while on site.	V, H, Th
20 Feb	Additional cat transferred to animal shelter	V, H, Th
21 Feb	No new events reported	V, H, Th
22 Feb	Poultry owner reached by telephone. Additional cat transferred to animal shelter.	
23 Feb	Two additional cats transferred to animal shelter. One cat euthanised (PCR-positive). Three of six cats in the shelter confirmed HPAI A(H5N1)-positive.	V, T, H, Th
24 Feb	Cleaning, disinfection and official sealing of poultry house	V, H, Th
25–28 Feb	Ongoing monitoring of exposed individuals	Th
1 Mar	Continued monitoring of exposed persons	Th
2 Mar	Notification according to §12 IfSG^a^ submitted	Th
3 Mar	One symptomatic veterinary authority staff tested; rapid antigen test negative. Additional PCR test (see below).	Th
4 Mar	Sterilisation of five cats at veterinary practice	T, Th
5–6 Mar	Continued monitoring of exposed persons	Th
5 Mar	Veterinary staff PCR negative	Th
9 Mar	Sterilisation of the sixth cat at the veterinary practice	T, Th

H: poultry holder; HPAI: highly pathogenic avian influenza; P: police officers; T: veterinarian/veterinary; Th: animal shelter staff; V: veterinary authority staff.

^a^ German Infection Protection Law for Notifiable Diseases. The contact person groups listed in the third column were identified as having had contact or exposure on the dates indicated in the table.

**Table 2:** Animals affected during the highly pathogenic avian influenza A(H5N1) outbreak, Sigmaringen, Germany, February 2026 (n = 21 poultry, n = 9 cats)

Animal species	Symptoms	Status	Number	HPAI A(H5N1) PCR result	Serology (ELISA)
Domestic cat	NA	Found dead on premises	1	Positive	Not done
Domestic cat	ARI, abscesses, general illness	Euthanised	1	Positive	Not done
Domestic cat	ARI, CNS	Euthanised	1	Positive	Not done
Domestic cats	One cat with mild ARI symptoms	Alive, transferred to animal shelter	6	3 positive^a^	Seropositive for H5-specific antibodies
Poultry		Found dead on premises	4	Positive	Not done
Poultry	NA	Remaining poultry culled	17	Negative	Not done

ARI: acute respiratory infection; CNS: central nervous system; HPAI: highly pathogenic avian influenza; NA: not applicable.

^a^ PCR-positive cats with the status “alive”: Cat 1: presented with respiratory symptoms, which were improving at the time of assessment. Cats 2 and 3: PCR-positive throat swab results, but asymptomatic. Cats 4, 5, and 6: status “alive"; PCR-negative throat swabs and asymptomatic.

By 23 February, the investigation revealed a further six cats on the premises; three tested PCR-positive for HPAI A(H5N1) (two asymptomatic, one mildly symptomatic). All cats were transferred to an animal shelter and isolated. During handling of the animals, shelter staff used personal protective equipment (PPE), including protective gowns, gloves, surgical masks and shoe covers. The local veterinary authority informed the Public Health Office about the involvement of the shelter on 24 February 2026, and investigations and consultations were conducted on the same day. The Public Health Office provided guidance to shelter staff on infection prevention and control measures, including the recommendation to use filtering face piece (FFP) 2 or FFP3 respirators instead of surgical masks. The local veterinary authority ensured that the poultry holding premises were cleaned and disinfected on 24 February, and the poultry house was sealed for 4 months in accordance with veterinary regulations.

## Laboratory investigations

Diagnostic investigations were conducted at the State Veterinary Diagnostic Laboratory in Aulendorf and confirmed by the Friedrich-Loeffler-Institute (FLI). Combined oropharyngeal/cloacal swabs from four chickens and brain tissue from the three cats tested positive for HPAI A(H5N1) by RT-qPCR with high viral loads. The quantification cycle (Cq) values in the central nervous system (CNS) of the cats ranged from 16.5 to 24.3. No oropharyngeal swabs from cats were tested. Analyses were performed employing a set of four RT-qPCR targeting the M gene as a generic influenza A-specific target as well as fragments of the H5 and N1 gene segments; pathotyping was also done by a RT-qPCR, targeting the endoproteolytic cleavage site of the H5 haemagglutinin gene of HPAIV clade 2.3.4.4b [6]. 

Serum samples, obtained from all surviving cats, were seropositive for H5-specific antibodies analysed by a commercial ELISA (ID Screen Influenza H5 Antibody Competition 3.0 Multi-species ELISA; Innovative Diagnostics, France), indicating infection even in animals without detectable viral RNA. The assay had been validated with sera from ferrets but not with canine and feline sera [7]. 

Sera from 11 of the 17 humans exposed to HPAI A(H5N1)-infected animals were analysed using a hemagglutination inhibition (HI) assay [8]. The assay was validated earlier in another study using human sera from a vaccination trial in which an inactivated influenza A(H5N8) vaccine was administered to human subjects. All participants tested negative before vaccination and developed antibodies against the listed strains following vaccination (data not shown). In the outbreak reported here, we used egg-propagated vaccine viruses and inactivated H5N1 and H5N8 viruses (Table 3). The HI assays employed turkey red blood cells; viruses were adjusted to eight haemagglutinating units for analysis. We detected no antibodies against HPAI A(H5N1), but observed reactivity against seasonal influenza viruses, indicating no serological evidence of zoonotic transmission.

**Table 3:** Overview of highly pathogenic avian influenza A(H5N1) viruses detected in serological analyses of exposed humans, Sigmaringen, Germany, February 2026 (n = 11)

Contact person	Strain and subtype							
	H1N1		H3N2		B/Victoria	H5		
	A/Victoria/ 4897/2022	A/Sydney/5/2021	10136/RV/2023	A/Thailand/8/2022	B/Austria/1359417/2021	A/Whooper swan /R65–2/06 H5N1	A/turkey/Germany/R2485 + 86/2014 H5N8	A/DE-SH/Reiherente/ AR8444/16_AV Ak 2341 H5N8
V	160	640	160	160	< 10	< 10	< 10	< 10
V	< 10	640	160	160	80	< 10	< 10	< 10
V	40	80	40	80	< 10	< 10	< 10	< 10
V	40	160	80	160	160	< 10	< 10	< 10
V	160	320	40	40	40	< 10	< 10	< 10
V	80	160	80	80	160	< 10	< 10	< 10
H	80	160	< 10	< 10	20	< 10	< 10	< 10
Th	2,560	5,120	80	80	1,280	< 10	< 10	< 10
Th	320	640	160	80	640	< 10	< 10	< 10
Th	5,120	5,120	320	320	80	< 10	< 10	< 10
P	320	1,280	80	160	80	< 10	< 10	< 10

< 10: negative; H: poultry holder; HPAI: highly pathogenic avian influenza; ID: identification number; P: police officers; T: veterinarian/veterinary; Th: animal shelter staff; V: veterinary authority staff.

Eleven of the 17 exposed individuals accepted the testing.

## Preliminary risk assessment

No suspected or confirmed human cases of HPAI A(H5N1) were identified. We assessed the risk for the general population as low, while occupationally exposed individuals had limited but increased risk. Detection of HPAI A(H5N1) in several cats indicates cross-species transmission and reminds us to maintain strict adherence to PPE during on-site outbreak investigations and all other activities on premises that may expose people.

The cats roamed freely on the property and probably had contact with infected poultry or contaminated environments. Veterinary investigations identified poultry carcasses with bite marks, suggesting infection through predation or scavenging. The affected poultry were free-ranging and may have had contact with wild birds potentially infected with HPAI A(H5N1). Poultry were fed outdoors, which may have attracted wild birds to the property. Detailed information on shared water sources and exact transmission routes was not available. The cats were primarily outdoor animals with limited contact with the poultry owner, who fed them outdoors.

## Public health response

Following notification of the outbreak, a coordinated One Health response involving veterinary and public health authorities was initiated. It involved identification of 17 exposed individuals, counselling on infection prevention, and symptom monitoring. We identified exposed individuals through on-site investigation, including interviews with the poultry owner and involved personnel as well as a review of activities on the premises and in related settings (e.g. the animal shelter). Risk stratification was based on the type and intensity of exposure. The use of PPE was uncertain and was not considered in risk stratification. Contact persons, i.e. exposed persons, were defined as individuals with direct exposure to infected animals, their secretions or contaminated materials or environments (n = 17). Due to uncertainty regarding adequate PPE use, all contacts (Table 4) were required to self-monitoring their symptoms for 7 days following the last exposure, based on a risk-adapted assessment and the expected incubation period of HPAI A(H5N1) [9]. Although longer monitoring periods (10–14 days) are generally recommended, we considered the shorter duration appropriate given the exposure setting with close follow-up of all exposed individuals. Diagnostic PCR testing was available at the State Health Office Baden-Wuerttemberg and the National Reference Centre for Influenza in case of symptom onset. 

**Table 4:** Human highly pathogenic avian influenza A(H5N1) contacts and monitoring measures, Sigmaringen, Germany, February 2026 (n = 17)

Group	Number	Exposure	Influenza vaccination	PPE use
Poultry holder	1	Contact with animals	Not vaccinated	None
Veterinary authority staff	7	Poultry holding inspection and cats	None vaccinated	None or partial (gloves) during early visits
Police officers	2	Site presence	Vaccinated^a^	None
Animal shelter staff	3	Contact with cats	Vaccinated^b^	Full PPE
Veterinarians^c^	4	Handling cats	One unvaccinated	One euthanised the first cat without PPE

HPAI: highly pathogenic avian influenza A(H5N1); PPE: personal protective equipment.

^a^ One police officer was vaccinated before the event. The other police officer was vaccinated post-exposure.

^b^ The animal shelter staff were vaccinated post-exposure.

^c^ One individual was unvaccinated; vaccination status was unknown for the remaining three. One individual euthanised the first cat without PPE; all subsequent contacts with infected or potentially infected cats from the outbreak site were conducted using appropriate protective measures.

Post-exposure antiviral prophylaxis and seasonal influenza vaccination were recommended for all contact persons. One veterinary staff member initiated post-exposure antiviral prophylaxis with oseltamivir following unprotected contact with poultry. Another exposed veterinarian developed respiratory symptoms. An initial rapid antigen test for influenza virus, severe acute respiratory syndrome coronavirus 2 and respiratory syncytial virus was negative. A nasal and throat swab collected 8 days after last exposure, at peak symptoms, was analysed by multiplex PCR panels for respiratory viruses, including influenza viruses and endemic coronaviruses, in two independent laboratories. Human coronavirus OC43 was identified in both laboratories. A detailed description of the PCR tests is provided in Oh et al [10].

## Discussion

In recent years, HPAI A(H5N1) viruses has caused substantial losses in poultry and wild bird populations across Europe [1,2]. Concurrently, increasing infections have been reported in carnivorous mammals, including domestic cats, highlighting an expanding host range and raising concerns about viral adaptation to mammalian hosts. Domestic cats are of particular concern because they have close contact with humans and are highly susceptible to these viruses [11-13].

This event highlights several important aspects: Firstly, early detection through veterinary surveillance, including animal welfare inspections, can facilitate timely identification of outbreaks in backyard poultry holdings. In this investigation, initial veterinary visits triggered further diagnostic testing after unexplained animal deaths were observed. Secondly, infections in mammals may occur during poultry outbreaks, and cats in particular can serve as indicators of substantial environmental virus circulation. The detection of HPAI A(H5N1) infection in several domestic cats on the affected premises demonstrates the potential for cross-species transmission under outbreak conditions. Such spillover events have increasingly been reported and point to an evolving host range of HPAI A(H5N1) viruses [3,4,14]. Serum samples, obtained from all surviving cats, were seropositive for H5-specific antibodies analysed by a commercial ELISA which suggests a close epidemiological link between poultry and feline infections, although the role of cat-to-cat transmission remains unclear. In this investigation, evidence of predation or scavenging (e.g. poultry carcasses with bite marks) suggests that infection in cats may have occurred through direct contact with infected birds. Finally, occupational exposure among first responders and veterinary personnel remains an important pathway for potential zoonotic transmission [1,4,14]. Several individuals involved in the initial response had unprotected contact with animals before confirmation of the outbreak.

During seasonal circulation of acute respiratory infections, clinical assessment of exposed individuals presenting with respiratory symptoms requires careful consideration, as symptoms may be caused by a wide range of circulating pathogens. Therefore, symptomatic individuals with occupational exposure should be assessed systematically and tested to exclude zoonotic influenza infection.

Seasonal influenza vaccination is recommended for people with occupational exposure or a potential risk of exposure to avian influenza viruses to reduce the risk of co-infection with human and avian influenza viruses and minimise the risk of viral reassortment. In this investigation, several veterinary staff had not received seasonal influenza vaccination. However, the majority of those tested had antibodies against seasonal influenza viruses, particularly influenza A(H1N1).

Although no human infections were identified, the event underscores the continued need for preparedness and integrated surveillance across animal and human health sectors. Previous reports, including a presumed cat-to-human transmission of low-pathogenic avian influenza A(H7N2) virus in 2016, demonstrate that infected cats represent a potential zoonotic source.

Our analysis has limitations: Firstly, systematic virological testing of all exposed individuals was not performed, as testing was limited to symptomatic persons, which represents a standard and pragmatic approach in this context. However, asymptomatic or subclinical infections may not have been detected. Secondly, we could not determine the precise timing of infection among animals as several animals had already died before the first veterinary inspection and the poultry holder was not immediately available. This limited our ability to accurately define the exposure period and assess the temporal sequence of transmission events. Blood samples were collected 21–28 days after potential exposure; however, the exact timing of exposure varied between individuals. Although the interval between exposure and sampling was at least 7 days—within the expected window for detectable antibody responses—no baseline samples were available. In the presence of detectable antibodies, recent seroconversion could not have been excluded. Thirdly, exposure assessments relied partly on retrospective reporting of PPE use. Fourthly, serological assessments of infection have several limitations. Antibody responses may be low or undetectable in asymptomatic or mildly infected individuals, and the timing of sample collection may not capture seroconversion. Finally, detailed genomic characterisation of the virus was not yet available.

## Conclusions

The detection of HPAI A(H5N1) in poultry and domestic cats in Sigmaringen district highlights ongoing zoonotic risks associated with HPAI outbreaks. Rapid interdisciplinary collaboration enabled early identification of the outbreak and implementation of targeted preventive measures. Overall, this outbreak illustrates the importance of timely coordination between veterinary and public health authorities, early risk assessment of exposed individuals, and implementation of preventive measures within a One Health framework. Appropriate use of PPE, diagnostic testing, and prompt reporting of suspected cases are essential to prevent unprotected exposures and facilitate coordinated investigations. Continued vigilance and coordinated One Health surveillance remain essential to mitigate zoonotic transmission.

## Data Availability

Outbreak investigation data such as epidemiological, serological data are available from the corresponding author upon reasonable request.
